# Fracture Strength of Tooth Roots Following Canal Preparation by Three Rotary File Systems: An In Vitro Study

**DOI:** 10.7759/cureus.57302

**Published:** 2024-03-31

**Authors:** Elvis Chiramel David, Azeem H, Emil Santhosh Mani, Sibin George, Indra Semwal, Akhila Raj R

**Affiliations:** 1 Department of Conservative Dentistry and Endodontics, MES Dental College, Perinthalmanna, IND; 2 Department of Conservative Dentistry and Endodontics, Uttaranchal Dental and Medical Research Institute, Dehradun, IND; 3 Department of Conservative Dentistry and Endodontics, Amrita School of Dentistry, Amrita Institute of Medical Sciences (AIMS), Amrita Vishwa Vidyapeetham, Kochi, IND

**Keywords:** tooth fracture, microcrack formation, neolix a1 file, protaper next, protaper universal

## Abstract

Background: Since the beginning of modern endodontics, there have been many concepts, strategies, and techniques for root canal preparation. A mind-boggling variety of files have developed for negotiating and shaping them throughout the years. Today's most secure, most effective, and simplest file system combines the most reliable design elements of the past with the latest technological advances to create the most effective file system. So, the need for the study is to evaluate the fracture strength of tooth roots following canal preparation by three rotary file systems: ProTaper Universal file system (Dentsply, USA), ProTaper Next file system (Dentsply Sirona USA), and Neolix A1 nickel-titanium (NiTi) file system (Orikam Healthcare India Pvt Ltd., New Delhi, India).

Method: Ninety human mandibular molars were selected for the study. Inclusion criteria include human mandibular first and second molars and teeth removed for routine clinical reasons, and intact apices were selected, excluding cases with root surface caries, root surface fissures, teeth with immature root apex, mesial canal fusion, extremely short roots, thin roots, or curved roots. All teeth were preserved in a solution of 10% neutral buffered formalin for two weeks and then transferred to distilled water for examination. The teeth were randomly divided into three groups. Access cavities were created, and working lengths were determined. Groups 1, 2, and 3 underwent shaping using ProTaper Universal, ProTaper Next, and Neolix A1 (NiTi) file systems, respectively, following guidelines. Canals were irrigated with sodium hypochlorite and ethylenediaminetetraacetic acid (EDTA) and were obturated up to the mid-root region with AH Plus sealer. To facilitate fracture testing, obturation was performed to distribute the load from the spreader to the canal wall. The EndoSequence and Quick-Fill obturation system were utilized to fill the apical half of the canal with gutta-percha material. After obturation, the distal root of each tooth was cut, while the mesial root was securely positioned in a putty material. A universal testing machine was employed for the fracture tests, operating at a cross-head speed of 1 mm/min. The machine was equipped with a D11 hand spreader tip, which was inserted into the root canal to make contact with the gutta-percha. Gradual force was applied to the root canal until a fracture occurred, at which point the force application was stopped. The amount of force required to cause the fracture was measured in newtons. Data were collected and recorded using IBM SPSS Statistics for Windows, Version 17.0 (Released 2008; IBM Corp., Armonk, New York, United States) and then transferred to Microsoft Excel for analysis. Descriptive statistics, mean, and standard deviation were used for continuous data. The fracture resistance of dental roots treated with three types of files was compared using a one-way ANOVA. Graphs were generated using Excel and Word. A significance level of p<0.01 was chosen.

Result: ANOVA indicated significant differences in mean fracture resistance: Neolix A1 (NiTi) (95.3 N) > NEXT (91.0 N) > universal (86.6 N), with a p-value of 0.004 (<0.001), confirming statistical significance.

Conclusion: The study concludes that the canal instrumented with Neolix A1 (NiTi) exhibits higher fracture resistance after canal instrumentation compared to ProTaper Next and ProTaper Universal.

## Introduction

The preparation of root canals, obturation procedures, and post-space preparation that cause excessive dentin removal will lead to enhanced root fracture vulnerability. The end goal of endodontic instrumentation is to remove tissue debris and a diseased dentinal inner layer while also providing an air and waterproof tight seal [1].

The primary challenge associated with root canal-treated teeth is vertical root fracture (VRF) [2,3]. It is a periodontal fracture that begins in the crown of the tooth, the apex of the root, or anywhere between these two points and extends to the periodontium [4]. There are many reasons for increased fracture susceptibility such as dehydration, excessive dentin loss, caries removal, access cavity preparations, effects of irrigating solutions, canal shaping techniques, excessive obturation pressure, overpacking of calcium hydroxide, preparation and placement of posts, procedures for retreatment, and tooth preparation for crown placement [5].

VRF poses a problem of difficult early clinical detection and care, which causes unwanted suffering for the patients and the dentists. Due to difficulties in identifying and diagnosing vertically broken teeth, clinical diagnoses such as VRF, split root syndrome, cracked tooth syndrome, and others have been developed [6-8]. Patients' symptoms might be mistaken for other conditions like ear pain, sinus infections, or blurred vision [9]. The primary radiographic indications consist of an expansion of the periodontal ligament on one or both sides of the root or bone loss in a particular tooth. Common clinical symptoms include mild pain or discomfort and swelling, while the primary clinical sign is the presence of a single pocket surrounding one tooth [10-12].

The primary reasons for VRF are commonly attributed to two factors: excessive loss of dental structure during endodontic instrumentation and the application of excessive obturation pressure [13]. The best approach to enhance fracture resistance involves preserving internal tooth structure and using smaller posts in teeth that have undergone endodontic treatment [14,15]. During canal instrumentation using nickel-titanium (NiTi) rotary/reciprocating files, momentary stresses in dentine are concentrated due to the contact and friction between the instrument and canal walls. This phenomenon leads to the formation of cracks, which have been observed and reported at various levels [16]. The type of rotary system used, as well as the technique employed during instrumentation, can influence the extent of dentin damage and subsequent fracture vulnerability. Understanding the impact of rotary systems on dentin structure is essential for minimizing the risk of VRF and preserving tooth integrity [17].

To improve the efficacy and efficiency of root canal instrumentation in endodontic procedures, Neolix is a cutting-edge rotary file system. Apart from its proficient layout, Neolix provides better cutting efficiency when compared to conventional rotary systems. This can be attributed to its superior materials and advanced manufacturing techniques. The files are made to be durable and sharp throughout the root canal process, even with the rigors of the instrumentation process [18,19]. Moreover, Neolix can be used with a variety of handpieces, obturation techniques, and rotary systems. This adaptability makes it possible for dental professionals to effortlessly incorporate into current clinical protocols, giving them the flexibility and adaptability to meet a wide range of patient needs. 

The purpose of this study is to delve into how different rotary systems used in endodontic procedures affect the structure of dentin and the development of cracks. The goal is to shed light on the fracture strength of tooth roots following canal preparation using three rotary file systems: ProTaper Universal file system (Dentsply, USA), ProTaper Next file system (Dentsply Sirona USA), and Neolix A1 nickel-titanium (NiTi) file system (Orikam Healthcare India Pvt Ltd., New Delhi, India).

## Materials and methods

Study methodology

The study used 90 human mandibular first and second molars, which were indicated for extraction due to poor periodontal prognosis and orthodontic reasons. Inclusion criteria include human mandibular first and second molars, teeth removed for routine clinical reasons, and intact apices. Significant root surface caries, root surface fissures, teeth with an immature root apex, mesial canal fusion, extremely short roots, thin roots, or curved roots were excluded. All teeth were preserved for two weeks in 10% neutral buffered formalin, then rinsed in distilled water before being examined. These 90 teeth were divided randomly into three groups (n=30). A straight-line access cavity was created in each tooth, and the working length was determined using a reference point. 

The formula for calculating the sample size is n=2x(Z_α/2_​+Z_β_​)^2^×σ^2​^, where Z_α/2_​ is the critical value for the desired level of significance (usually 1.96 for a 95% confidence level), Z_β_​ is the critical value for the desired power of the test (typically 0.84 for 80% power), σ^2^ is the population variance, and δ is the minimum clinically significant difference to be detected.

Study groups

Groups were as follows: Group 1: ProTaper Universal file system (Dentsply, USA) and Group 2: ProTaper Next file system (Dentsply Sirona USA). A size 25.06 tapered file was used for shaping the coronal third, followed by a size 20.06 tapered file till reaching the working length, and a size 25.06 tapered file was again used for shaping the apical third for both ProTaper Universal file system and ProTaper Next file system.

Group 3: Neolix A1 (NiTi) file system (Orikam Healthcare India Pvt Ltd., New Delhi, India). Size 25, taper at the tip: 12%, length: 15 mm, rectangular cross-section. The canals of all the teeth were filed following the manufacturer's procedural guidelines. Irrigation with a solution containing one percent sodium hypochlorite (Deor) and 17% ethylenediaminetetraacetic acid (EDTA, Avueprep-Dental Avenue) was carried out for each canal. After instrumentation, the canals were irrigated with normal saline. Finally, canals were rinsed with distilled water using a 2 ml disposable syringe. To seal the canals up to the mid-root region, AH Plus (Dentsply Sirona India) was used. During fracture testing, obturation was employed to evenly distribute the load from the spreader to the canal wall. The apical part of the canal was filled with gutta-percha using the Endodontic and Quick-Fill obturation techniques.

For the testing process, each tooth's distal root was removed with a microtome saw, and the mesial root was secured in place. Before setting, the apical root ends of the mesial root were vertically embedded into acrylic resin blocks measuring 4 mm in depth. To prepare for testing, a putty material was used around the root, and it was allowed to harden for 30 minutes. A steel conical tip with a diameter of 0.5 mm, tapered at a 60° angle, was affixed and positioned in alignment with the center of the canal orifice, running parallel to the longitudinal axis of each specimen.

Fracture testing

**Figure 1:**
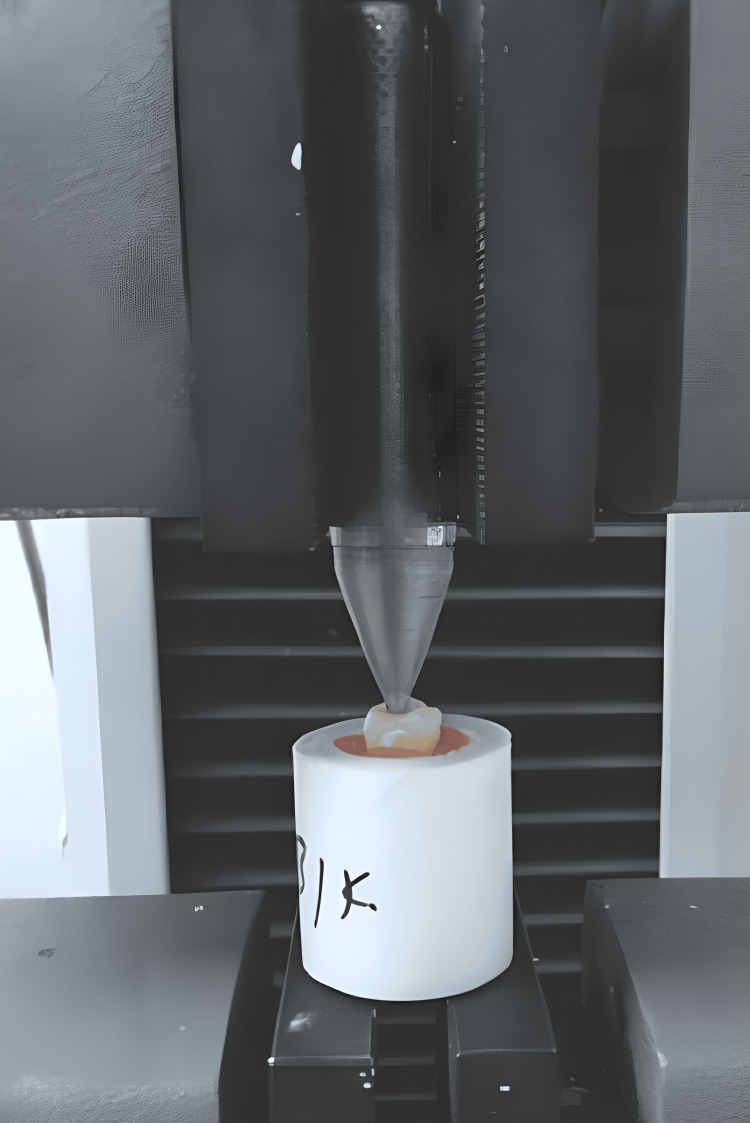
Testing machine for the fracture resistance.

The roots were then subjected to fracturing, and the fracture load was measured in newtons using a universal testing machine with a crosshead speed of 1 mm/min. The force applied with the hand spreader within the root canal via the gutta-percha ceased as soon as the fracture occurred (Figure 1).

Statistical analysis

The data collected during the study were recorded using IBM SPSS Statistics for Windows, Version 17.0 (Released 2008; IBM Corp., Armonk, New York, United States), which was then loaded into a Microsoft Excel data sheet for evaluation. Continuous data were described using the mean and standard deviation. The study aimed to compare the fracture resistance of dental roots after they were treated with three different types of files. To perform this comparison, a one-way ANOVA test was employed. Graphical representations of the data, such as bar diagrams and box plot graphs, were created using MS Excel and MS Word.

To determine the statistical significance of the results, a p-value of 0.01 was considered, indicating the probability that the findings were true. Both MS Excel and IBM SPSS Statistics for Windows, Version 17.0 were utilized as statistical tools for data analysis. 

## Results

The statistical analysis using one-way ANOVA revealed that the mean fracture resistance following canal instrumentation was highest for Neolix A1 (NiTi) (95.3 N), followed by NEXT (91.0 N), and the lowest for universal (86.6 N). The calculated p-value of 0.004 (<0.01) indicated that the study's results were statistically significant.

The Neolix A1 (NiTi) file system also has the lowest standard deviation, indicating less variability in the data compared to the other two systems. The ANOVA test was conducted and found to be significant at the 99% confidence level (p-value <0.01), suggesting that there are statistically significant differences in fracture resistance among the three file systems (Table 1).

**Table 1:** Comparison of fracture resistance between three file systems. NiTi: nickel-titanium.

Variable	N	Mean	Standard deviation	Standard error	99% Confidence interval for mean		F	p-value
					Lower bound	Upper bound		
Universal	30	86.67	14.30	2.61	81.33	92.01	6.023	0.004
Next	30	91.06	7.43	1.36	88.28	93.83		
NiTi	30	95.31	4.37	0.80	93.68	96.94		
Total	90	91.01	10.17	1.07	88.88	93.14		

## Discussion

Two types of root fractures can happen in permanent teeth: transverse intra-alveolar root fracture, typically occurring in anterior teeth due to trauma, and VRF, which can occur in both anterior and posterior teeth as a result of traumatic occlusion or iatrogenic operations [19]. Tamse et al. conducted a study to assess clinically and radiologically the vertically fractured endodontically treated teeth both before and after extraction. Among the teeth examined, the upper second premolars and mesial roots of mandibular molars were found to be the most commonly fractured ones [20]. Endodontic instrumentation has progressed in tandem with technological improvements during the previous few decades. Motorized file systems presently in use have rotating blades and flutes with an increasing taper and a solid metal core. It causes active cutting, and dentine removal is more in comparison with other files [21].

According to a study by Pawar et al. [22], samples instrumented by the self-adjusting file exhibited better fracture resistance when compared to rotary ProTaper NEXT and single reciprocating WaveOne file systems when filled with gutta-percha and epoxy-based sealer. In this research, the fracture strength of mesiobuccal tooth roots of mandibular first and second molars was compared after being instrumented with three different continuous rotary file systems representing various generations in the development of rotary endodontics. The file systems have been compared to see how susceptible the tooth root is to fracture after instrumentation with these file systems and subsequent loading with a D11 spreader tip. Instruments composed of the single-file system and ultra-elastic NiTi alloy are used in these systems, which are thought to bend well even in curved canals. They can easily access the apical foramen within the first files due to their great flexibility, thereby reducing the number of files being used.

VRF develops when tiny cracks within the tooth's structure gradually extend over time. These microcracks emerge on the root's dentin due to the excessive stress exerted during endodontic instrumentation. It is important to note that fractures don't happen immediately after the root canal procedure [21]. The rotary files are linked to 18%-60% of microcracks created during biomechanical preparation, while the reciprocating files have three times more impact [22]. The vertical fracture resistance is reduced by over 30% after shaping and cleaning. These cracks can become regions of increased stress concentration and progressively propagate the cracks to root canal surfaces [22]. In endodontic treatment, the point where the instrument comes into contact with the dentinal wall is referred to as the midsection. These regions can experience elevated and temporary stress concentrations in the dentin, potentially leading to nerve and tooth damage. The existence of such pressures in these roots is likely to exacerbate dentinal imperfections and raise the risk of cavities when performing dental procedures [23].

The ProTaper Next rotary files have a unique design in which the center of rotation and center of mass are offset when the file is rotating. This allows for more space cross-sectionally, which allows for better cutting, loading, and coronally auguring of debris. There were fewer microcracks recorded with these files, causing fewer microcracks due to the reduced screwing effect because of their swaggering motion (alternating taper) and thus diminished file-dentin contact [7]. Moreover, due to their composition of M-wire alloy, these files exhibit greater flexibility when compared to nickel-titanium alloy (NiTi) files [24]. However, the enhanced rotational movement of ProTaper Universal files may lead to elevated stress concentrations in the walls of the root canal. These files also possess designs that generate higher tensile and compressive stresses, which can result in increased stress levels at the apex of the root dentin and create high-strain components on the external surface of the root [25].

The main purpose of the NeoNiti file system is to create a funnel-shaped configuration in the root canal. This is achieved by utilizing a technique that generates a rough and abrasive surface, which ultimately speeds up the process of root canal preparation. The files themselves possess a non-homothetic rectangle cross-section and are subjected to suitable heat treatment, which effectively improves their flexibility [26].

Furthermore, advanced design feature contributes to a reduction in microcrack formation and is associated with improved fracture resistance. Similarly, the unique characteristics of Neolix A1 (NiTi), single-file system, including its non-homothetic rectangle cross-section and suitable heat treatment, contribute to its enhanced flexibility and, potentially, its ability to reduce the risk of microcracks and fractures. These findings underscore the significance of continuous advancements in endodontic file technology to minimize the potential risks associated with root canal procedures and improve the long-term prognosis of endodontically treated teeth.

The limitations of this study include its laboratory-based nature, which may not fully replicate the complexities of clinical settings. The use of extracted teeth, although common in endodontic research, does not account for the living biological environment within the oral cavity, potentially affecting the results. Additionally, the exclusion of specific root conditions and the standardized treatment protocols employed may not cover the diverse clinical scenarios encountered in real-world practice. The choice of a specific file system might not fully represent the wide variety of files available in contemporary endodontics, potentially limiting the generalizability of the findings. Furthermore, while fracture resistance was assessed, the study did not consider other clinical factors that could influence the longevity and success of endodontically treated teeth, such as the quality of coronal restorations and occlusal forces. Finally, the study's focus on immediate fracture resistance may not fully predict long-term clinical outcomes.

## Conclusions

Throughout the investigation, the authors carefully assessed the average fracture resistance after canal instrumentation, taking into account the number of files used in each file system. The findings revealed that the Neolix A1 (NiTi) file system, comprising a singular innovative file, exhibited the highest mean fracture resistance among the tested groups. In contrast, the Next file system, consisting of multiple files, demonstrated a moderately lower fracture resistance, followed by the Universal file system, which employed a conventional approach involving multiple files as well. The emphasis on the Neolix A1 (NiTi) system being a single-file approach is crucial, as it underscores the potential benefits of simplifying endodontic procedures while optimizing outcomes. 
